# Learning from usability testing of an arts‐based knowledge translation tool for parents of a child with asthma

**DOI:** 10.1002/nop2.369

**Published:** 2019-09-10

**Authors:** Mandy M. Archibald, Shannon D. Scott

**Affiliations:** ^1^ Helen Glass Centre for Nursing University of Manitoba Winnipeg MB Canada; ^2^ Faculty of Nursing Level 3 Edmonton Clinic Health Academy University of Alberta Edmonton AB Canada

**Keywords:** arts, asthma, children, nurses, nursing

## Abstract

**Aim:**

Digital, art‐ and story‐based resources can be viable and engaging knowledge translation strategies in health care. Understanding the usability of these approaches can help maximize their impact. The aim of this work is to understand what aspects of ‘My Asthma Diary’, an art‐based digital knowledge translation tool for parents of children with asthma, has an impact on usability.

**Design:**

Sequential explanatory mixed methods pilot study.

**Methods:**

Eighteen parents of children with asthma reviewed ‘My Asthma Diary’ in a paediatric emergency department and completed a usability questionnaire. Follow‐up interviews were conducted with five parents and analysed with qualitative description.

**Results:**

We identified four themes which complemented the quantitative results: (a) the eBooks are relatable and mirror personal experience; (b) the digital format is convenient and easy to navigate; (c) the narrative structure aids learning; and (d) the narrative and illustrations are synergistic. We summarize core usability considerations for subsequent research and creative knowledge translation tool development in other contexts.

## 1 INTRODUCTION

Persons are arbitrators of their own presence in the world and they should have the last word… texts must always return to and reflect the words persons speak as they attempt to give meaning and shape to the lives they lead. (Denzin, 2014, p. 4)

How can creative methods of representing research be optimized to improve research engagement and optimize illness self‐management? Creative knowledge translation (KT) strategies are increasingly regarded as viable means of supporting this engagement by providing research evidence in palatable, meaningful and comprehensible formats (Archibald, Caine, & Scott, 2014). Creative KT involves approaches that leverage non‐academic modes of communication, such as digital media (e.g. infographics, social media) and the arts (e.g. storytelling, theatre), to target outcomes traditionally of interest to KT, such as attitude, knowledge and behaviour change (Alberta Addiction & Mental Health Research Partnership Program, 2015). Arts‐based KT is therefore a subset of creative KT and is defined as approaches incorporating the arts to disseminate and communicate knowledge, while offering opportunities for embodied learning and participant engagement (Archibald et al., 2014). Arts‐based KT can leverage multiple learning styles, emotional understanding and forms of knowledge beyond the explicit, propositional knowledge characteristic of didactic teaching approaches (Archibald, Caine, & Scott, 2017; Archibald et al., 2014). These attributes have utility in various health and illness contexts and may be particularly pertinent to enduring illness contexts such as childhood asthma because of the experiential knowledge that individuals bring to each healthcare encounter and management scenario (Archibald, Hartling, Caine, Ali, & Scott, 2018).

Most asthma management, the most common chronic disease of childhood, occurs outside of healthcare settings (Deis, Spiro, Jenkins, Buckles, & Arnold, 2010; Nicholas, Dell, Fleming‐Carroll, & Selkrik, 2009). This requires parents to undertake a great extent of day‐to‐day care. Consequently, ensuring that parents are equipped to manage asthma day‐to‐day is integral to improving childhood asthma outcomes. However, research continues to show that parents have pervasive unmet information needs around childhood asthma (Archibald, Caine, Ali, Hartling, & Scott, 2015; Archibald & Scott, 2014; McMullen et al., 2007). This points to shortcomings in how research evidence on asthma management has been mobilized and communicated to parents (i.e. often through standard information sheets or verbal information provision only) (Archibald et al., 2015). These shortcomings contribute to poor asthma control and avoidable burdens on the family (e.g. frequent exacerbations, missed school and work) and healthcare system (e.g. high use and cost) (Berg, Anderson, Tichacek, Tomizh, & Rachelefsky, 2007; Coffman, Cabana, Halpin, & Yelin, 2008; McMullen et al., 2007).

## 2 BACKGROUND

### 2.1 Process of eBook development: ‘My Asthma Diary’

In response to the need for more complex educational materials that reflect parental experience, we created ‘My Asthma Diary: Coping with Childhood Asthma’, a 28‐page art and story‐based eBook developed in response to parental information needs identified from an interpretive descriptive study (Archibald et al., 2015). The process of developing ‘My Asthma Diary’ involved multiple stages, which we report on in previous manuscripts (Archibald et al., 2015, 2018; Archibald & Scott, 2014) and summarize briefly here. In stage one, we conducted a state of the science literature review (Archibald & Scott, 2014) to identify domains of parental information needs to inform a semi‐structured interview guide and to identify whether a subsequent primary research study was needed. These domains included asthma basics (e.g. basic pathophysiology and knowledge of triggers including environmental modification), treatment modalities (e.g. short‐ and long‐term medications), coping (including self‐efficacy) and medical expectations (e.g. when to seek medical assistance) (Archibald & Scott, 2014). In stage two, we conducted an interpretive descriptive study of asthma information needs with 21 parents of a child with asthma (Archibald et al., 2015). In this study, we identified four predominant information needs (recognizing severity, acute management and inhaler use, prevention vs. crisis orientation and knowing *about* asthma) and two mediating factors (beliefs about the nature of asthma, interactions with healthcare providers) (Archibald et al., 2015). We then used the parental stories, experiences and knowledge need to structure the narrative for ‘My Asthma Diary’ and used an interdisciplinary, collaborative process to develop four asthma eBook prototypes (Archibald et al., 2018). These four asthma eBooks were then presented to a unique sample of parents for usability testing (current manuscript).

**Figure 1:**
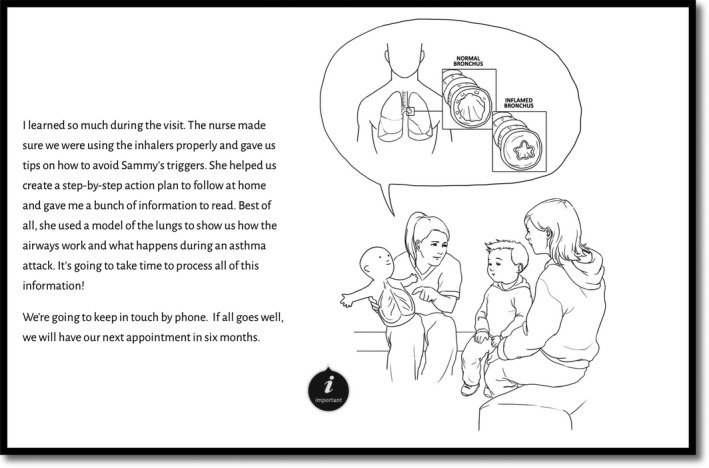
Example page from eBook prototype 1. Black and white line drawing version with sans‐serif font

**Figure 2:**
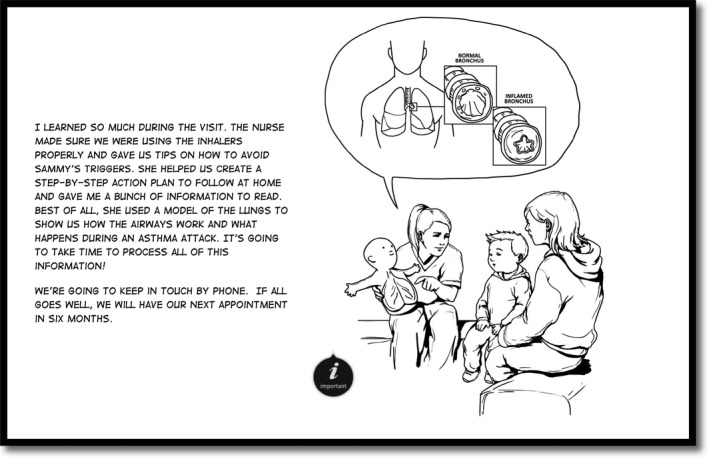
Example page from eBook prototype 2. Graphic novel style black and white line drawing version with comic font

**Figure 3:**
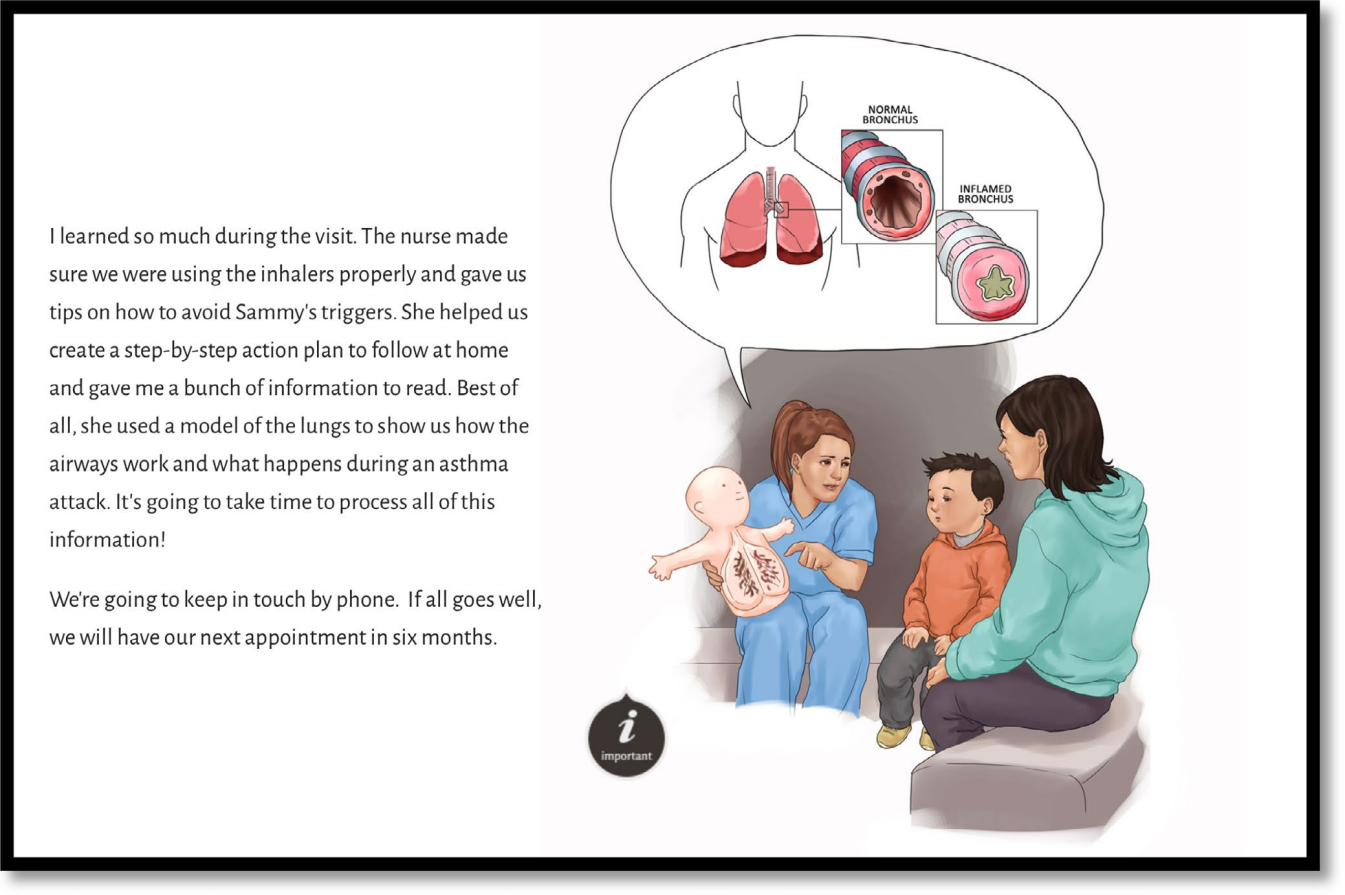
Example page from eBook prototype 3. Blended colour illustration with sans‐serif font

**Figure 4:**
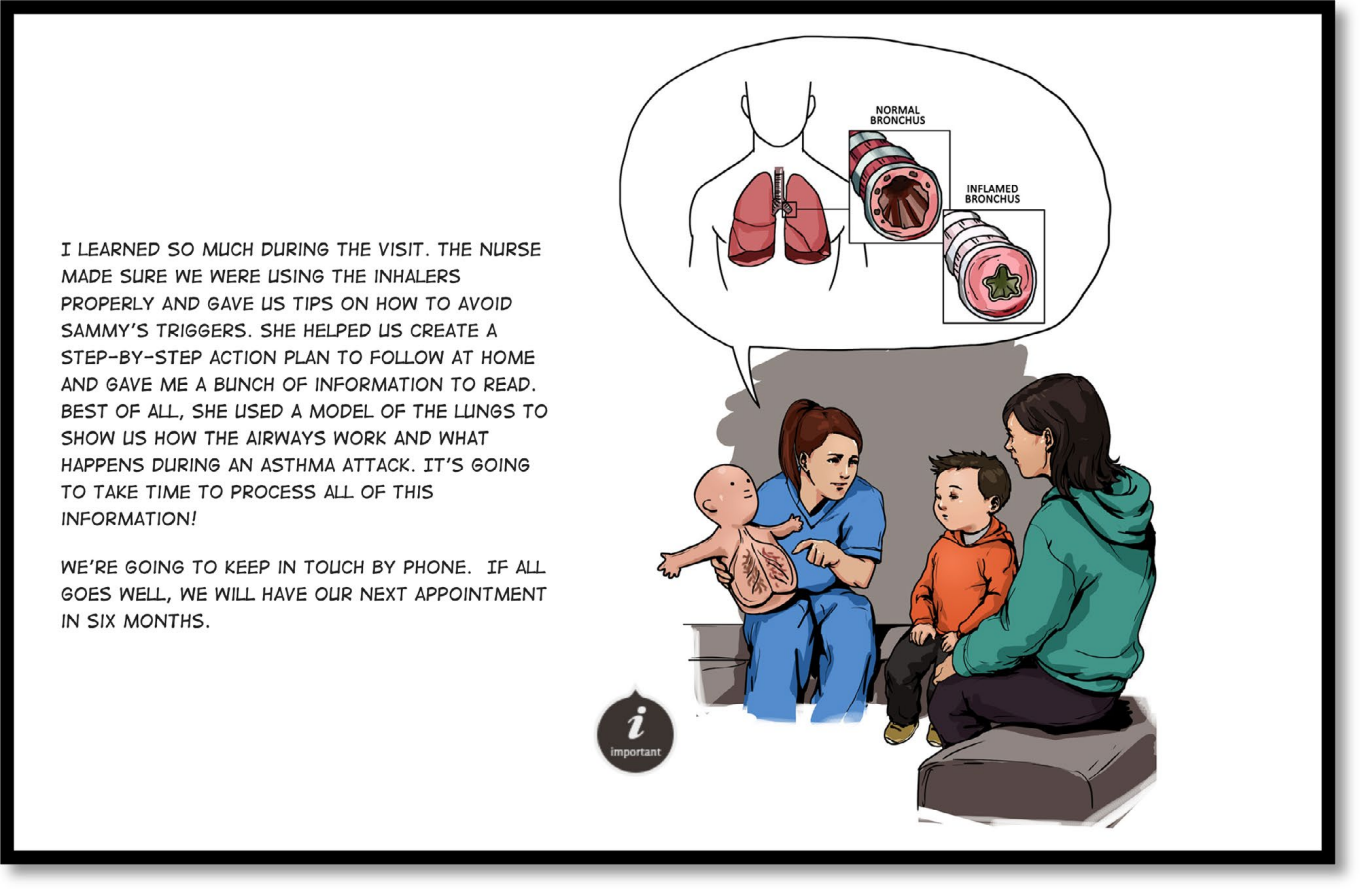
Example page from eBook prototype 4. Graphic novel style colour illustration with comic font

Each eBook prototype contained the same narrative, structure and evidence‐based content and differed in the aesthetic style of presentation (i.e. illustration style, text font and colour) as visible in Figures 1, 2, 3, 4. We detail the development of the eBook in a separate publication (Archibald et al., 2018) and report here on the usability of these resources.

### 2.2 Usability testing

Usability testing is a common practice in health informatics—the array of assessment and communication activities integrating technology with a human interface (Bastien, 2010). The purpose of usability testing is to determine whether a developed product meets its intended purpose and, as such, is crucial to any user‐centred design process (Bastien, 2010). Central to usability testing are questions pertaining to effectiveness (e.g. does the produce do what it intended?), efficiency (what is the resource investment for the system to be used as it was intended?), satisfaction (positive responses from end‐users) and errors encountered (Alshaman & Mayhew, 2009; Bastien, 2010; Stinson et al., 2010). Although examples of user‐based usability assessment (i.e. approaches that involve direct end‐user participation) are readily available in the health literature, most revolve around website development (Alshaman & Mayhew, 2009). More research is needed on what makes Web‐*hosted* (digital) arts‐based KT resources usable, in part because of the emergent status of such approaches (Archibald et al., 2014). Such data will be useful to guide similar strategies in different populations and contexts, particularly given the growing popularity of arts‐based KT approaches across disciplines.

Methods of assessing usability are guided by the intricacy of the KT strategy and end‐user characteristics. Kastner, Lottridge, Marquez, Newton, and Straus (2010) conducted usability evaluations of three components of a clinical decision support tool for osteoporosis disease management. Using a standardized in‐person process with a trained moderator and a think‐aloud procedure, the authors collected and analysed video, audio and statistical data to iteratively inform the tool's revision. Like Ben‐Zeev et al. (2013) whose usability testing for a smartphone self‐management system for schizophrenia focused on ease of system use, decisions regarding the importance of real‐time feedback were made in reference to the complexity of the delivery system and related content. Stinson et al. (2010) conducted usability testing of an online self‐management program for adolescents with juvenile idiopathic arthritis. Using an iterative 2‐cycle user‐based approach, they conducted qualitative semi‐structured interviews and observation methods to detect user performance and revise the self‐management prototype. This cyclical approach, inclusive of observation, was necessary given the complexity of the ‘12‐module interactive multi‐component treatment protocol’ (Stinson et al., 2010, p. 3), which consisted of over 310 content pages, forums and surveys, among other features.

Conversely, Hartling et al. (2010) used an iterative qualitative approach when developing storybooks for childhood croup. The authors employed user‐centred design principles to understand end‐user perceptions of the stories and storybook prototypes and revised these according to the feedback obtained through participant review and focus group interviews. By focusing on the presentation, interest, style and clarity of the croup stories, the authors were able to maximize the ‘accuracy, credibility and relevance’ (p. 9) of the storybooks and to identify considerations that align with established usability principles (e.g. identify clear goals and purpose) (HHS.gov, n.d).

Although usability procedures used in the health sciences vary extensively, they should reflect evidence‐based practices in user‐centred design and draw on the plethora of international standards that have been developed over the past 25 years (Bevan, 2001). For instance, the U.S. Department of Health and Human Services provides best practices for Web‐based content, design and usability, including research‐based guidelines on design process (e.g. give useful content, establish user requirements) and user experience (e.g. increase Web credibility). When adapted, these principles can inform the development and usability evaluation of digital, arts‐based KT strategies.

Applying usability principles to the development and refinement of KT tools can enhance their relevance, uptake and impact (Graham, Kothari, & McCutcheon, 2018). Yet little research exists on the usability of arts‐based KT approaches, despite growing use of these approaches across disciplines. In this study, we sought to understand which aspects of ‘My Asthma Diary’ have an impact on its usability, to contribute to the growing evidence base of arts‐based KT tools and help inform future effectiveness studies. In this paper, we report on a usability study examining parent's perceptions of ‘My Asthma Diary’—a parent‐driven, arts‐based KT eBook developed for parents of children with asthma (Archibald et al., 2018) and discuss how the lessons learned can be applied to other creative KT endeavours with different populations.

## 3 DESIGN

We conducted a sequential explanatory mixed methods usability pilot for the purposes of complementarity (i.e. elaborating on and clarifying quantitative results using qualitative methods) (Greene, Caracelli, & Graham, 1989) using a complementary strengths stance (Morse, 2003). We drew from two traditions: technology development, where a basic usability assessment using quantitative methods was conducted (Ancker, Chan, & Kukafka, 2009; HHS.gov, n.d), and a generic qualitative approach, where a deeper exploration of the meaning of the quantitative responses and additional perceptions of the eBooks were elicited.

Determining optimal sample size for usability testing is the subject of long‐standing debate. Bastien (2010) identifies that four to five participants may uncover approximately 85% of usability issues encountered with an electronic interface. While notable support for this approach exists (e.g. Kastner et al., 2010), others purport that asking, ‘how many users is needed’ is perhaps the wrong question to ask. Given that usability testing is seeking to uncover mismatches between the user interface and the users' mental model, an alternative method of considering sample size relates to the number of tasks an end‐user is being asked to complete (Straub, 2007). As very few unique tasks (e.g. access information icons, navigate sequentially through story) were involved in navigating through ‘My Asthma Diary’, we hypothesized that a quantitative sample of 16–20 participants and a qualitative sample of 4–7 participants would sufficiently uncover most usability issues.

## 4 METHODS

### 4.1 Quantitative

Parents of a child with asthma who presented to one of two emergency departments in an urban centre (population: approximately 880,000) were approached on a convenience basis to discuss the study. Eligible parents were English speaking and identified as a primary caregiver for a child with asthma or asthma‐related concern. Parents were invited to review one of four eBook prototypes (i.e. prototype three), complete a paper‐based 27‐question usability questionnaire and consent to a follow‐up interview. Parents were fully informed about the study prior to providing verbal consent to participate.

The questionnaire included four pre‐ and post‐test visual analog scale (VAS) measures (i.e. level of asthma knowledge, comfort with asthma knowledge, confidence in managing asthma day‐to‐day, confidence in managing asthma exacerbations) anchored at 0 (‘not at all’) and 10 (‘very much’), 15 descriptive VAS measures specific to three dominant usability constructs (i.e. effectiveness, efficiency, satisfaction) and four open‐ended questions. The VAS was developed based on recognized usability constructs and additional measures adapted for arts‐based KT (e.g. relatability**)**.

### 4.2 Qualitative

Consenting parents were contacted for a semi‐structured telephone interview. The interview guide was developed to reflect usability constructs having an impact on ease of use in the user‐centred design literature, to explore quantitative findings and to identify ‘incidents’. Like Kastner et al. (2010), we defined an incident as a problem or error that either: (a) halted the typical function or navigation within the eBook (i.e. critical incident); or (b) did not inhibit completion (i.e. general incident). Incidents were categorized by type and content.

Consenting parents were provided an online link to the asthma eBooks (Table 1) and time to review the books prior to commencing the interview. Interview questions moved from general to specific, beginning with an exploration of parental perceptions of the eBook prototype three, which parents had previously reviewed. After exploring general usability questions, parents were asked the following specific questions regarding their perceptions of the prototypes: (a) Do you prefer any one version of the eBook and if so, which one and why? (b) Can you rank the eBooks according to your preference? (c) What do you like and dislike about each version? Interviews were audio‐recorded.

**Table 1:** Overview of eBook prototypes

Prototype #	Illustration type	Colour	Text font
1	Line drawing	Black and white	Sans‐serif font
2	Line drawing, graphic novel style	Black and white	Comic font Sans‐serif font
3	Blended colour illustration	Colour	
4	Graphic novel style	Colour	Comic font

Qualitative and quantitative integration occurred at the levels of sampling, data collection and analysis. Qualitative participants were sampled from the quantitative arm, enabling exploration of participant responses obtained on the VAS measures. At the level of data collection, the questions from the semi‐structured interview guide were developed to explore, confirm or contest the quantitative findings. Analytic integration is discussed under the analysis heading.

## 5 ANALYSIS

We used IBM SPSS Software to analyse the quantitative data using non‐parametric (Wilcoxon signed‐rank test), parametric (paired *t* test) and descriptive statistics. Statistical significance was set at .05. Open‐ended questions were content analysed. Qualitative data were analysed inductively, guided by qualitative description. Audio recordings were listened to repeatedly, detailed notes and analytic memos were made, and data were loosely grouped thematically. Themes were revised and categories regrouped as analysis progressed. Rigour was enhanced through detailed memos and through team meetings where emerging findings were discussed and rigorously questioned. Problems encountered while using the eBooks were classified as critical or general incidences (i.e. usability issues that do or do not impair use), and participants were asked for suggestions to improve the eBooks function in relation to occurring incidences. We then used a sequential mixed analysis approach to analysing and integrating qualitative and quantitative data (Teddlie & Tashakkori, 2009). Data were analysed in sequence, and then, qualitative and quantitative findings were reviewed in reference to one another, to interpret the overall usability of the eBooks.

## 6 RESULTS

### 6.1 Quantitative

A trained research assistant collected data between March and November 2014. Twenty‐one parents were approached. Eighteen parents of children with asthma reviewed the eBook and completed the usability questionnaire, resulting in a response rate of 81.8%. No missing data on the VAS measures were encountered. Open‐ended questions were infrequently completed.

Parents, on average, reported that it took 9 min and 24 s to read the eBook (*SD* 4.00, range 5–20), which they regarded as an appropriate length (mean* = *8.44, *SD* 1.21, range 6.57–9.86). Descriptive statistics illustrate positive ratings on constructs related to effectiveness, efficiency and satisfaction. Outliers were encountered; for instance, participants occasionally provided very low ratings on the enjoyment, contribution of drawings to information and quality of information subscales. Open‐ended responses and follow‐up qualitative interviews suggest that participants may not have accessed the information icons (e.g. buttons that when activated, delivered additional information) embedded throughout the tool, relying exclusively on the storyline to receive information. One participant who provided a low enjoyment rating found the story was ‘scary and intimidating’ for first time parents dealing with asthma (Table 2).

**Table 2:** Descriptive statistics of usability findings

	Mean	*SD*	Range of values
Effectiveness	8.07	1.45	5.21–9.71
Information clarity	8.27	1.46	4.57–9.86
Ease of understanding	9.04	1.07	6.43–9.79
Remember information	8.50	1.41	4.93–9.79
Contribution of drawings to information	6.97	2.71	0.04–9.86
Contribution of story to information	8.10	1.83	4.29–9.86
Enjoyment	7.05	2.61	0.10–7.05
Satisfaction with format	8.29	1.66	3.71–9.93
Quality of information	7.71	2.64	0.15–9.93
Ease of finding information	7.77	2.01	3.50–9.93
Story reflects experiences	7.12	2.90	0.86–9.79
Relate to visual characters	5.20	2.93	0.07–9.5
Relate to story	7.46	2.35	2.71–9.86
Relate to written characters	7.03	2.18	3.07–9.86

Given the small quantitative sample and lack of random sampling, we conducted pre–post evaluations of knowledge gain (*p* = .001), confidence in managing asthma day‐to‐day (*p* = .055) and confidence in managing asthma exacerbations (*p* = .021) using the related‐samples Wilcoxon signed‐ranks test (i.e. non‐parametric paired *t* test equivalent). We ran paired *t* tests to obtain data on mean differences and standard deviations. Paired *t* tests revealed significant improvements in knowledge (*t*(17) = 3.09, *p* = .007) between pre (mean* = *6.81, *SD* = 1.98) and post (mean* = *7.78, *SD* = 1.45) measures; and significant improvements in confidence in managing asthma exacerbations (*t*(17) = 2.33, *p* = .032) between pre (mean* = *7.66, *SD* = 2.41) and post (mean* = *7.94, *SD* = 2.45) measures.

### 6.2 Qualitative and mixed analysis

Of eight parents who consented for follow‐up, five (two fathers, three mothers) completed a telephone interview. All self‐identified as the child's primary caregiver; had been managing asthma for a period ranging from 1–8 years; and had educational attainments at the high school (*n = *1), technical school (*n = *3) and university (*n = *1) levels. Interviews lasted an average of 36 min (range: 24–41.83 min). Iterative data collection and analysis confirmed that participants were identifying few usability issues; it appeared unlikely that further incidents would be encountered through additional data collection.

Parents had an overwhelmingly positive response to the eBooks. They consistently reported that the information was clear (mean = 8.27, clarity of information measure) and relevant, the digital format was appealing and easy to use (mean = 9.04, ease of understanding measure), and the storyline (mean = 7.46) and characters (mean = 7.03) were relatable and engaging. Parents unanimously reported satisfaction with the eBook (mean* = *8.29), including its format, content, method of delivery and length. Four themes were identified from the qualitative analysis and are presented here as narrative statements: (a) the eBooks are relatable and mirror personal experience; (b) the digital format is convenient and easy to navigate; (c) the narrative structure aids learning; and (d) the narrative and illustrations are synergistic. These themes are discussed in reference to affiliated quantitative measures and open‐ended questionnaire responses.

#### 6.2.1 The eBooks are relatable and mirror personal experience

It talks to you…and everyone can find a way to relate to it. (participant 3)

Participants unanimously reported that the eBooks were highly relatable (mean = 7.46), regardless of whether the storyline reflected precisely their experiences. The emotional connection made possible through the telling and reliving of the fictional mother's story was a source of relatability for many participants and the uncertainty surrounding the asthma trajectory often mirrored participants' experiences. Delivering information through a first‐person narrative enhanced participants' emotional connection and provided emotional validation (e.g. participants 4, 5)—the eBook was regarded as more personal than other methods of delivering information (e.g. pamphlets) and enabled participants to ‘put yourself in their shoes… it's a real person… it's real information… it was very comforting’ (participant 3).

All participants emphasized how the eBook was appropriate for use with their child with asthma. The engaging illustrations and story made the eBook useful for reading with their child (e.g. participants 1, 2, 3). Participant 3 expressed, ‘you can't put a medical document in front of a four‐year old’ and ‘here's a story and… she relates to this!’. Participants one and four credited the eBook to opening a dialogue about asthma with their child. Parents recognized the need for children to learn about his/her asthma and the eBook was seen as a useful tool to facilitate this learning.

Participants expressed that the storyline reflected the reality of living with childhood asthma, thereby increasing its relatability. Specifically, participants noted that the ending of the story, wherein the central character (a 6‐year‐old boy) achieves asthma control but does not outgrow asthma, was particularly salient, believable (participants 1–5) and hopeful (participant 3). As participant 1 emphasized, the ‘growing out of asthma’ discourse was prevalent among her family and friends; the concluding sentiments in the eBook provided a needed reflection of the reality of living with asthma. For others, the turning point signified by the asthma clinic referral (participant 4), or the seasonal variations in asthma (participants 1, 5) present in the narrative were particularly reflective of parents' experiences.

One participant identified that the use of multicultural names and characters effectively ‘reflects the communities we are living in’ and signified inclusivity (participant 5). While other participants did not explicitly identify this as a factor having an impact on relatability, it may have influenced the high quantitative (mean = 7.46) and unanimously positive qualitative relatability ratings among the diverse participant sample. Namely, all participants, including the fathers, were able to relate to the story, despite the narrator being a mother. As participant 2 explained, he was still able to relate to the narrative because stories need not be interpreted literally and because having a mother as the central character may indeed reflect a reality of the caregiving experience.

#### 6.2.2 The digital format is convenient and easy to navigate

The digital format was convenient. It gives instant access anytime I need it. (participant 3)

All participants were satisfied with the digital delivery format, consistent with the quantitative results (mean* = *8.29). Participants identified that convenience, portability and accessibility were augmented through the digital delivery. Digital delivery was also linked with ease of use for participants, a finding supported by the quantitative ‘ease of finding information’ measure, (mean* = *7.77). Although each participant used the digital format in different ways, all participants used the navigational arrows to advance through the eBook. However, if and how participants accessed the ‘important’ information icons and external resource links varied, with few participants making use of the external resources (e.g. links to additional URLs).

Participants enjoyed the convenience and portability of accessing the eBook in any setting. As participant 2 expressed, he enjoyed being able to access the eBook while waiting for medical care, which was regarded as a good use of his time. Similarly, in one open‐ended response, a parent indicated that the eBook was ‘the perfect length’ to keep her occupied while awaiting further treatment. Participants felt it would be easier to refer back to an eBook than paper‐based information, and some identified specific content that was most useful to refer back to (e.g. emergency kit, list of triggers).

Most participants preferred the digital delivery to a paper‐based format (participants 1, 2, 4, 5). One participant (participant 3) expressed nostalgia for paper‐books but concurrently stated that the eBook was a highly useful and convenient modality for asthma education. Similarly, in an open‐ended response, one participant requested paper copies so that the information could be re‐visited. Overwhelmingly, participants recognized the digital format as appropriate and reflective of the current digital era: a belief that ‘everyone has a computer’ (participant 4) or would use their hand‐held device to access information (participant 2) rendered the eBook a contemporary educational modality. For one participant (participant 5), the digital approach enhanced the eBooks' credibility: ‘you see some of the brochures and you just know that they've been there for 20 years and are stale and out of date, not that reliable… because the format was digital you think, okay this is new, or newer, than a dusty old brochure or pamphlet and likely easier to update’.

#### 6.2.3 The narrative structure aids learning

The storyline helped understand the parent's perspective… and triggers your memory. (participant 4)

Participants found the story to be an effective communication approach because it helped organize information in a manner that ‘flowed’. The narrative gave structure to information that otherwise would be a series of facts. Parents expressed satisfaction with the quality of information provided; embedding information within a sequential narrative allowed participants to follow and make sense of the information (participants 1–5). In this way, the narrative ‘made the information manageable’ (participant 3), improved information recall (open‐ended response, p. 5) and enhanced the eBooks' ease of use, thereby supporting the quantitative findings.

Participants identified that the flow of information was appealing (participant 1, 2, 3), which supports the quantitative findings (mean* = *7.05, enjoyment in reading eBook measure). As participant 3 expressed, ‘Putting the important information into a story, makes me feel better… it's not just some medical information booklet that you get and you read it and your bored and you don't understand half of what they're saying…’. An important component of this appeal was that the storyline situated the information in the context of family life: ‘this wasn't just a generic asthma brochure but was a story of a family. That reinforced the seriousness of the condition’ (participant 5).

The narrative was structured according to the four seasons to reflect seasonal variations in asthma in Canada. This was meaningful for participants, mirrored participants' own uncertainty and variable trajectories and for one participant, ‘emphasized the need to be consistent year round’ (participant 1). In addition to the seasons, participants often identified the discussion of triggers (participant 1, 4, 5), specifically pertaining to the family pet, as reflective of their own experiences (participant 5). When the storyline did not reflect the specifics of the participant's experience, the eBook was regarded as useful in ‘getting the conversation going’ (participant 1).

#### 6.2.4 The narrative and illustrations are synergistic

The narrative and the visuals work together. (participant 4)

The illustrations enhanced the eBooks' aesthetic and aided in its functionality. Participants expressed that the illustrations contributed to comprehension, engagement and overall appeal (participants 1–5). They referred to the interplay between the story and illustrations, a finding supported by participants' quantitative ratings (mean* = *6.97, contribution of drawings to information; mean* = *8.1 contribution of story to information; mean* = *9.04, ease of understanding), which suggests that the narrative and visual components of the eBook work together synergistically. Indeed, participants reinforced this notion through statements such as ‘the storyline triggers your memory… and the pictures help us think’ (participant 4).

The illustrations reinforced and complimented the eBook content. Participants emphasized how the illustrations improved information clarity and made the research‐based information more comprehensible (participants 1–5); the amount of detail in the illustrations reinforced the key learning points (participants 1, 3 and 4) and at times, helped re‐orientate participants to what their children were experiencing. A particularly effective illustration in this respect was a visual rendering of constricted bronchial tubes (Figures 1, 2, 3, 4), that, as participant 4 expressed ‘I forget what it looks like for her tubes… you just think, ‘the lungs’… this shows what she's really dealing with… what she is breathing out of’.

The participants enjoyed the illustrations and style, which helped relate to and engage with the information provided. As one participant expressed, ‘if there were no drawings, or illustrations, [the eBook] would be very boring’ (participant 2). While four out of five participants related to the illustrations and one felt the depictions were ‘dead‐on’ (participant 1), participant 4 found the mother looked exceedingly worried; she expressed concern that this could worry her child if they read the eBook together. Interestingly, the same participant felt that the eBook could emphasize the experience of uncertainty more thoroughly. Overall, the qualitative results support and expand on the quantitative findings: the story contributed more to the information than the illustrations, but both were integral to the effectiveness, efficiency and satisfaction of engaging with the eBook.

### 6.3 Prototype preference

Color is more attractive and captures our imaginations. (participant 2)

Participants unanimously preferred the coloured versions of the eBooks (prototypes 3, 4) to the black and white versions (prototypes 1, 2). The black and white versions were seen as ‘unfinished’ (participant 5), less relatable and the illustration details (i.e. Figures 1, 2, 3, 4) were less discernable. Prototype 1 was the least appealing prototype for all participants, followed by prototype 2—the darker lines of prototype 2 were seen to enhance its appeal.

Two of the four participants who ranked the eBooks favoured the ‘eye‐catching’ (participant 1) and ‘familiar’ (participant 4) graphic novel style of prototype four, while the remaining two preferred the illustration style of prototype three. Participants were divided into whether the comic‐like depiction was engaging, or whether it appeared juvenile.

Participants had mixed perspectives about font. Participant 1 preferred the boldness and ease of reading of the comic font (prototypes 2, 4), where participant 2 found the comic font difficult to read, stating, ‘there is no sign of where words start or stop’. Two participants commented on how the text font ‘matched’ the illustration styles for all prototypes; the sans‐serif font was regarded as a ‘softer, more conversational’ match for the diary (participant 5).

### 6.4 Incidents

We encountered two general incidents. First, 80% of participants did not notice the Table of Contents icon in the lower corner of each page. We classified this as a general incident since it did not impede participants' ability to navigate sequentially through the eBook. Participants suggested various strategies to improve icon visibility (e.g. moving the icon to the upper corner, or adding colour or animation). Second, two participants identified that the ‘important’ icons (i.e. icons that can be clicked on to receive additional information) could be more noticeable. Three additional open‐ended responses suggested that the ‘important’ icons were not accessed. Participants suggested describing these icons and their importance in the eBooks' introduction, or adding colour or animation to the icons.

### 6.5 Limitations

There were limitations to our study. We did not conduct real‐time, in‐person usability testing in a naturalistic environment, which could have revealed different usability issues (Alshaman & Mayhew, 2009). Determining sample size for usability testing is a continued debate in the field with little sign of resolution (Bastien, 2010), and the qualitative sample was limited to those parents consenting to follow‐up. However, the lack of occurrence of new usability issues attested to appropriate sample size for the study purpose, recognizing that we may have encountered additional usability issues with a larger sample. The quantitative arm did not have a control group and was not randomly sampled, which would have been a more critical issue if assessing effectiveness of the intervention, rather than the usability effect as designed.

## 7 DISCUSSION AND CONCLUSION

Participants in our study found the combined use of story and art facilitated engagement, understanding and appeal, thereby supporting the use of a multimodal dissemination approach. Effective stories create an emotional connection with readers (Kirkpatrick, Ford, & Castelloe, 1997) and enable vicarious re‐experience—the capacity to experience and feel through representation (Barone & Eisner, 2012). Visual art can move beyond the confines of language to elicit and evoke different and deeper understandings. Evoking an emotional response is central to the aesthetic experience and to learning more generally; as such, balancing aesthetics with the integrity of the research data are integral consideration with arts‐based KT (Archibald et al., 2014, 2018; Hartling et al., 2010).

Our findings reflect favourably on using a user‐centred design process. Parents who accessed the ‘important’ information icons expressed satisfaction with the quality of research‐based information provided. Similarly, Hartling et al. (2010) reflected on the challenge of creating aesthetically appealing yet informative stories with generalizable appeal for parents of children with croup. The authors obtained critical feedback from parents on the relatability and usefulness of the storybooks through focus group interviews at the prototype stage and individual interviews during the evaluation stage (Hartling et al., 2010). While various forms of research can be used to inform arts‐based KT tools (e.g. literature review for cartoon‐based dissemination, Lafreniere, Hurlimann, Menuz, & Godard, 2014), maximizing participant involvement throughout development and evaluation may enhance pertinence and appeal and foreseeably improve effectiveness. Others who have developed knowledge tools for families have found through usability testing that a user‐centred design is fundamental to creating useful and meaningful products (Shoup et al., 2015; Taddio et al., 2013).

There are useful, transferable lessons gleaned from usability testing of arts‐based and digitally delivered KT tools. Usability testing provides essential data on whether creative KT tools achieve their purpose. Using an asynchronous approach, we were able to determine whether ‘My Asthma Diary’ met its purpose while simultaneously identifying key considerations for others interested in developing creative educational materials in other populations and contexts. These considerations are summarized in Table 3.

**Table 3:** Summary of usability considerations and key findings

Decision point	Preference	Justifications & considerations
Narrative voice	1st Person	Improved relatability over 3rd person narrative
	Mother	Primary narrator reflected common reality of asthma management
Narrative message	Realistic yet hopeful	Narrative needs grounding in data to promote relatability To reduce fear, a message of hopefulness, problem‐solving and resilience is critical
Text font and size	Easy to read	Certain text fonts reduce readability by making it difficult to determine where words stop and start Difficulties enhanced with English as second language participants
Illustrations	Colour	Viewed as a finished, aesthetic product
	Non‐specific ethnicity	Promotes relatability across diverse ethnic backgrounds
	Engaging	Useful to share with child
Structure	Context specific (e.g. four seasons in Canada)	Improved relatability, acceptability, meaning‐making and information recall Selected structure mirrored seasonal variations in asthma and mirrored cognitive structure of marking time in Canadian context
Time to complete	<10 min	Appropriate with concurrent activities (child care) and for participant attributes (busy parent) Good use of time during hospital visit
Visibility of table of contents	Very noticeable	Consider different colour or animation for icon to promote visibility
Delivery method	Digital	Regarded as contemporary, up to date and accessible

Central to usability considerations is an understanding of end‐user needs (e.g. understood through formative research), fit with purpose (e.g. does it communicate research in a meaningful and comprehensible way?), efficiency (e.g. is the tool an appropriate length, particularly when considering user attributes and contexts of application) and satisfactory display (e.g. is using the tool enjoyable?). Regardless of the creative modality employed, consideration to user‐centred design principles is necessary to ensure that KT tools are functional and appealing (Bastien, 2010). This is particularly pertinent given the emergent status of arts‐based KT tools, the lack of existing data on how attributes unique to arts‐based KT tools have an impact on their use, and the extensive resources (personnel, financial, time) required for their development (Archibald et al., 2014).

## CONFLICT OF INTEREST

The authors have no conflicts of interest to report.

## ETHICAL APPROVAL

The Research Ethics Board at the University of Alberta granted approval for this study. Informed consent was obtained prior to parent's involvement in the quantitative study arm. Parents consented to follow‐up qualitative interviews following completion of the questionnaire. Verbal consent was obtained prior to completing the telephone interviews.
